# Advances in Pharmaceutical Analytical Technology

**DOI:** 10.3390/molecules30102155

**Published:** 2025-05-14

**Authors:** Alexis Oliva

**Affiliations:** Departamento de Ingeniería Química y Tecnología Farmacéutica, Facultad de Farmacia, Universidad de La Laguna, 38200 Tenerife, Spain; amoliva@ull.edu.es

The rapid development of new analytical methods has led to advances in many areas, from the formation of new pharmaceutical analytical technologies to the quality control of new drugs at different stages of enhancement and the search for new dosage forms [1,2,3].

In essence, quality by design (QbD) can be interpreted as a strategy for the maximization of time and cost savings. Optimizing the safety, efficacy, and quality of a drug requires a thorough understanding of the formulation and manufacturing process at every stage of progression. The full implementation of the QbD approach in the pharmaceutical field has still not been realized, despite the undeniable benefits of the QbD approach and the widespread information on the regulatory expectations of QbD.

This Special Issue contains thirteen manuscripts covering the latest advances in pharmaceutical analytical techniques, focusing on applying QbD principles to the development of analytical methods [4,5,6,7,8]. These principles promote the use of DoE and MLR strategies [9,10]. All the articles in this Special Issue perfectly capture these principles.

The papers collected here represent contributions from leading research groups around the world. They provide insights into the current state of and future directions in the field of analytical methods. For example, Klawitter et al. [Contribution 3], developed a new analytical platform for the assessment of the metabolism and the pharmacokinetics of the antineoplastic drug gemcitabine, which is currently under investigation. In Wang et al. [Contribution 2], the advancement of a cell-based in vitro assay for the biological activity of thyroid-stimulating hormone (TSH) was achieved. Some examples of practical applications also stand out, such as a new, environmentally friendly, high-throughput microwell spectrophotometric assay (MW-SPA) for the determination of three selective serotonin reuptake inhibitors (SSRIs) in their pharmaceutical forms [Contribution 11]. Stimac et al. [Contribution 10] analyzed the suitability of multi-detection SEC as a tool for monitoring molecular processes during antibody (IgG)–horse radish peroxidase (HRP) conjugation reactions and analyzed its plausibility for final product quality control. Peña et al. (Contribution 9) designed the first gas chromatography–mass spectrometry (GC-MS) method to simultaneously quantify 17α-ethinylestradiol (EE) and drospirenone (DP) in contraceptive formulations according to the International Council for Harmonization (ICH) Q2(R1) guidelines.

All research articles in this Special Issue demonstrate significant progress in the development and application of analytical methods for quantifying drugs in complex matrices [Contributions 1,3,7], pharmaceutical preparations [Contributions 2,7,9,11], or biological samples [Contributions 5,6,8,10], with applications in different analytical fields. After the satisfactory advancement and optimization of the analytical method, a comprehensive validation method according to the ICH-Q2 (R1) guidelines and QbD principles is performed to verify that the method is suitable for the intended purpose [Contributions 1–11].

This Special Issue also contains two review articles, where valuable insights into recent improvements in detection methods are provided. Lis-Cieplak et al. [Contribution 12], conducted a comprehensive discussion of the main strategies to improve the analytical efficiency of pyrrolizidine alkaloid (PA) determination via mass spectrometry (MS). PAs are toxic compounds that occur naturally in certain plants, but there are many secondary pathways that lead to the PA contamination of other plants, including medicinal herbs and herbal foods, which poses a risk of intoxication in humans. This review examines various detection means, focusing on liquid chromatography (LC) methods combined with mass spectrometry (MS) detection and using different types of analyzers characterized by varying sensitivities. The authors provide valuable guidance for future developments in MS detection by critically analyzing the advantages and limitations of each method.

The second review by Chen et al. [Contribution 13] explains the state of research on the chemical composition, pharmacology, and quality control of F. ferulaeoides, a traditional indigenous Chinese medicine with significant pharmacological activity, including insecticidal, bactericidal, and anti-tumor properties. This work aims to provide a reference for quality assessment and resource use in anticipation of the application of this product in the industry.

Both research papers and reviews are valuable references for researchers involved in the progression and application of analytical techniques, with a particular focus on the pharmaceutical field, and they demonstrate excellent scholarship and provide comprehensive overviews of their respective fields.
